# Endoplasmic reticulum proteostasis impairment in aging

**DOI:** 10.1111/acel.12599

**Published:** 2017-04-23

**Authors:** Gabriela Martínez, Claudia Duran‐Aniotz, Felipe Cabral‐Miranda, Juan P. Vivar, Claudio Hetz

**Affiliations:** ^1^ Center for Geroscience, Brain Health and Metabolism Santiago Chile; ^2^ Biomedical Neuroscience Institute Faculty of Medicine University of Chile Santiago Chile; ^3^ Program of Cellular and Molecular Biology Institute of Biomedical Sciences University of Chile Santiago Chile; ^4^ Center for Integrative Biology Universidad Mayor Santiago Chile; ^5^ Instituto de Ciências Biomédicas Universidade Federal do Rio de Janeiro Rio de Janeiro Brasil; ^6^ Buck Institute for Research on Aging Novato CA 94945 USA; ^7^ Department of Immunology and Infectious diseases Harvard School of Public Health Boston MA 02115 USA

**Keywords:** aging, endoplasmic reticulum, endoplasmic reticulum stress, protein misfolding disorders, unfolded protein response

## Abstract

Perturbed neuronal proteostasis is a salient feature shared by both aging and protein misfolding disorders. The proteostasis network controls the health of the proteome by integrating pathways involved in protein synthesis, folding, trafficking, secretion, and their degradation. A reduction in the buffering capacity of the proteostasis network during aging may increase the risk to undergo neurodegeneration by enhancing the accumulation of misfolded proteins. As almost one‐third of the proteome is synthetized at the endoplasmic reticulum (ER), maintenance of its proper function is fundamental to sustain neuronal function. In fact, ER stress is a common feature of most neurodegenerative diseases. The unfolded protein response (UPR) operates as central player to maintain ER homeostasis or the induction of cell death of chronically damaged cells. Here, we discuss recent evidence placing ER stress as a driver of brain aging, and the emerging impact of neuronal UPR in controlling global proteostasis at the whole organismal level. Finally, we discuss possible therapeutic interventions to improve proteostasis and prevent pathological brain aging.

## Introduction

As the world population gets older, dementia emerges as a major public health issue worldwide, particularly in middle‐ and middle‐to‐high‐income countries. The prevalence of dementia increases exponentially with age, affecting 5–10% of people over 65, and about 50% of people over 85. In 2011, dementia was estimated to affect 35.6 million people around the world, and it is expected to reach about 135 million by 2050 (Brayne, 2007; World Health Organization and Alzheimer's Disease International 2012). Reduced cognitive function is a common trait present in elderly individuals, which correlates with substantial alterations to functional synapses and normal neuronal physiology at the cellular and molecular level (Leal & Yassa, 2015). Accordingly, a significant percentage of aged individuals will manifest some sort of dementia in the form of a collection of neurodegenerative diseases, transposing the line between normal aging (healthspan) to pathological brain aging (Brayne, 2007). Recently, several interconnected processes have been defined as the hallmarks of aging, where substantial alterations to cellular proteostasis is proposed as one of the major pillars of aging (Lopez‐Otin *et al*., 2013; Kennedy *et al*., 2014).

The proteostasis network is decomposed into different subpathways highly conserved across evolution and comprehends a collection of mechanisms related to protein synthesis, folding, trafficking, secretion, and degradation distributed in different compartments inside the cell (Balch *et al*., 2008; Powers & Balch, 2013). The main players of this network include chaperones and foldases, the ubiquitin–proteasome system, the autophagy pathway, the heat‐shock response, the unfolded protein response (UPR), the integrated stress response, the endoplasmic reticulum (ER)‐associated degradation machinery (ERAD), the mitochondrial UPR, and the mechanisms controlling redox balance (Balch *et al*., 2008). Those processes are dynamic and tightly coordinated by quality control systems to avoid proteotoxicity and ensure that unfolded or misfolded proteins do not accumulate into cytotoxic aggregates (Labbadia & Morimoto, 2014). Various pathological conditions affecting the nervous system share common molecular features despite presenting different clinical manifestations, highlighting the presence of abnormal protein aggregates in the brain of affected individuals (Walker *et al*., 2015). These age‐related diseases are classified as protein misfolding disorders (PMDs) and include Alzheimer's disease (AD), Parkinson's disease (PD), amyotrophic lateral sclerosis (ALS), Huntington's disease (HD), prion‐related disorders (PrDs), among others (Soto, 2003). Importantly, one of the main nodes of the proteostasis network involved in aging and PMDs is the UPR and the occurrence of abnormal levels of ER stress. Recent advances in model organisms have uncover the significance of the UPR to the control of global proteostasis during aging, where the nervous system has a central role in monitoring alterations in the health of the proteome to adjust the capacity of the cell to cope with ER stress in various peripheral tissues. Here, we discuss new concepts illustrating the functional relevance of the UPR to organismal aging across species and its significance as a risk factor to develop neurodegenerative diseases.

## The unfolded protein response

The ER is the main site for the synthesis and folding of around one‐third of the total proteome of a cell (Braakman & Bulleid, 2011). Considered a key component of the proteostasis network, ER‐located proteins regulate folding and quality control through the activity of multiple chaperones, foldases, and co‐factors that assist the folding of nascent proteins as well as degradation pathways, thus preventing abnormal protein aggregation and resultant proteotoxicity (Ellgaard & Helenius, 2003; Kourtis & Tavernarakis, 2011; Hetz *et al*., 2015). Stressful stimuli such as hypoxia (Badiola *et al*., 2011), nutrient deprivation (Szegezdi *et al*., 2006), increased protein oxidation (Santos *et al*., 2009), and disturbance of the secretory pathway (Badr *et al*., 2007) may lead to an excessive accumulation of misfolded proteins at the ER, a process termed ER stress (Walter & Ron, 2011; Hetz, 2012). To cope with ER stress, a highly conserved signaling pathway is engaged known as the UPR (Wang & Kaufman, 2016). The UPR is initiated by the activation of at least three types of stress sensors including inositol‐requiring enzyme‐1 (IRE1), PKR‐like ER kinase (PERK), and activating transcription factor 6 (ATF6). IRE1 catalyzes the unconventional splicing of the mRNA encoding X‐box binding protein‐1 (XBP1) (Yoshida *et al*., 2001; Calfon *et al*., 2002; Lee *et al*., 2002), resulting in the expression of an active transcription factor called XBP1s that controls the expression of a cluster of genes related to folding and quality control mechanisms (Hetz *et al*., 2011). Additionally, IRE1 also degrades several mRNAs, ribosomal RNAs, and microRNAs through a process known as regulated IRE1‐dependent decay (RIDD), having an impact on different processes including inflammation and apoptosis (Maurel *et al*., 2014). IRE1 also engages distinct stress pathways, including JNK and NF‐κB, through the binding of adapter proteins (Hetz *et al*., 2015). Activation of PERK leads to the phosphorylation of the eukaryotic translation initiation factor 2 alpha (eIF2α), which in turn inhibits translation decreasing protein influx into the ER (Ron & Walter, 2007). Paradoxically, some mRNAs, including activating transcription factor 4 (ATF4), are differentially translated, leading to the upregulation of genes related to redox homeostasis, amino acid metabolism, autophagy, and apoptosis control (Harding *et al*., 2003; Ye & Koumenis, 2009; B'Chir *et al*., 2014). Moreover, PERK activation has been shown to modulate the activity of nuclear factor erythroid 2‐related factor 2 (NRF2) and forkhead box O (FOXO), linking this pathway to the antioxidant response, insulin responsiveness, and autophagy (Chevet *et al*., 2015). Under ER stress conditions, ATF6 translocates to the Golgi apparatus where it is cleaved releasing ATF6f, a cytosolic active form. ATF6f exerts its action at the nuclear level as a transcription factor regulating genes associated with ERAD, in addition to enhancing XBP1 mRNA transcription (Yamamoto *et al*., 2007). Importantly, the control of gene expression by the UPR depends on the cellular context and the stimuli considering that UPR transcription factors can interact with other proteins to drive specific responses, in addition to be regulated by several post‐translational modifications (Hetz *et al*., 2015). Under chronic ER stress, the UPR triggers apoptosis through different mechanisms that involve the upregulation of CHOP, the induction of oxidative stress, exacerbated RIDD, upregulation of pro‐apoptotic components of the BCL‐2 family, among other mechanisms (Tabas & Ron, 2011; Urra *et al*., 2013). Thus, under conditions of ER stress, the UPR reprograms the cell toward adaptation, sustaining cell function or the engagement of cell death programs to eliminate irreversibly damaged cells.

## ER proteostasis and aging in simple model organisms

**Figure 1:**
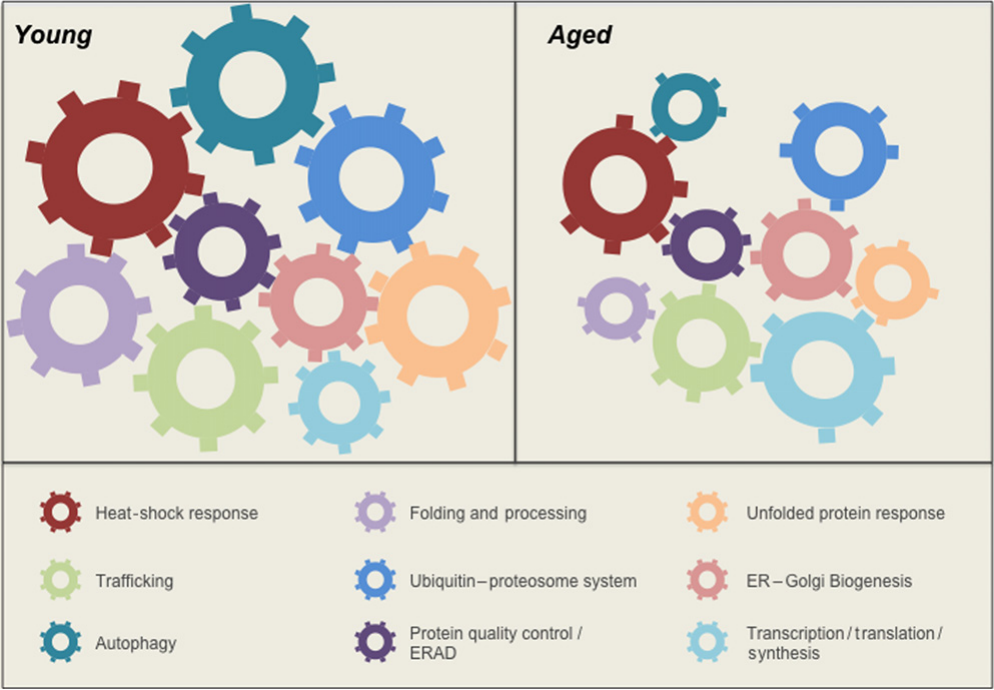
The buffering capacity of the proteostasis network decreases with aging. The aging process is directly associated with a range of specific alterations in distinct components of the proteostasis network. Such alterations disrupt the healthy functioning of cell and may contribute to the emergence of disease.

During aging, organisms gradually accumulate intracellular aggregates composed by misfolded proteins, an event that is associated with a prominent decline in the buffering capacity of the proteostasis network and a consequent decrease in tissue and cellular function (Fig. 1) (Taylor & Dillin, 2011; Triplett *et al*., 2015). Several studies in model organisms have uncovered the significance of UPR signaling to the aging process, associated with protection against proteotoxicity (Ben‐Zvi *et al*., 2009; Labunskyy *et al*., 2014). For example, caloric restriction has been used as a major strategy to prevent the adverse effects of aging on healthspan (Riera & Dillin, 2015). In yeast, this intervention correlates with increased expression of HAC1, the functional homologue of XBP1s (Choi *et al*., 2013). Remarkably, genetic ablation of HAC1 abrogates the lifespan extension conferred by caloric restriction (Choi *et al*., 2013). Other studies indicated that the deletion of distinct UPR‐target genes impact replicative lifespan in yeast, a process dependent on the Ire1p/HAC1 axis (Labunskyy *et al*., 2014). Furthermore, genetic modifications to improve the activity of the UPR enhance replicative lifespan in *Saccharomyces cerevisiae* (Cui *et al*., 2015).

Studies in *Caenorhabditis elegans* have revealed a fundamental role of the UPR in adjusting organismal proteostasis during aging through a neuronal control. Exposure of *Caenorhabditis elegans* to pharmacological inducers of ER stress indicated that the ability of aged worms to respond is significantly reduced compared with young animals (Ben‐Zvi *et al*., 2009). Interestingly, the same observation was reported when animals were stimulated with heat shock (Ben‐Zvi *et al*., 2009), suggesting global proteostatic defects during aging. Enforced expression of heat‐shock factor 1 (HSF‐1) or the FOXO‐transcription factor DAF‐16 restores proteostasis of aged worms (Ben‐Zvi *et al*., 2009). Loss‐of‐function studies in *Caenorhabditis elegans* demonstrated that lifespan extension conferred by XBP1 expression is dependent on insulin/IGF‐1‐FOXO signaling, a classical pathway associated with aging (Henis‐Korenblit *et al*., 2010; Douglas *et al*., 2015). Importantly, lifespan extension was only achieved through the parallel interaction between XBP1 and FOXO‐transcription factor DAF‐16, which acts in conjunction to genes related to longevity (Henis‐Korenblit *et al*., 2010). According to these observations, Taylor and Dillin demonstrated that the selective overexpression of Xbp1s in neurons or intestine strongly reverts the age‐related susceptibly to ER stress stimulation (Table 1) (Taylor & Dillin, 2013). Remarkably, the overexpression of XBP1s in neurons has a strong impact on lifespan extension, augmenting animal survival up to 30%. A recent study also indicated that loss‐of‐function mutations in distinct subunits of translation initiation factor eIF‐3 confer a 40% extension in the lifespan of *Caenorhabditis elegans* through a DAF‐16‐dependent and UPR‐independent pathway (Cattie *et al*., 2016), suggesting that different nodes of the proteostasis network significantly contribute to aging in worms.

**Table 1:** Genetic manipulation of the unfolded protein response (UPR) affecting the aging process in different model organisms. Examples of genetic manipulation to UPR components in different species that modifies lifespan with modified genetic backgrounds (*Alg12*,* Bst1*,* Pmt‐1*,* or Daf‐2* mutants) or under metabolic stimulus (caloric or dietary restriction)

Model organism/species	Genetic modification of UPR	Effect on aging	Stimulus/genetic background	References
*Saccharomyces cerevisiae*	Decreased expression of *Hac1*	Decreased lifespan	Caloric restriction	Choi *et al*. (2013)
	Deletion of *Hac1* or *Ire1p*	Decreased lifespan	Deletion of *Alg12* and *Bst1*	Labunsky *et al*. (2014)
	Deletion of *Hac1* or *Ire1p*	Decreased replicative lifespan	Deletion of *Pmt‐1*	Cui *et al*. (2015)
*Caenorhabditis elegans*	Deletion of *Ire1* or *Xbp1*	Decreased lifespan	Deletion of *Daf‐2*	Henis‐Korenblit *et al*. (2010)
	Deletion of *Atf6*	No effect on lifespan	Deletion of *Daf‐2*	
	Overexpression of *Xbp1* and *Daf‐16*	Extended lifespan, through the expression of longevity genes	Deletion of *Daf‐2*	
	Neuronal overexpression of *Xbp1s*	Increased lifespan through cell‐nonautonomous mechanism	Basal levels	Taylor & Dillin (2013)
*Drosophila melanogaster*	Deletion of *Perk* on intestinal stem cells	Increased lifespan	Basal levels	Wang *et al*. (2015)
	Deletion of *Ire1* or *Xbp1*	Decreased lifespan	Dietary restriction	Luis *et al*. (2016)

Studies in flies have also defined contributed to define the relevance of the UPR to the aging process. Intestinal stem cells in *Drosophila melanogaster* promote a regenerative response upon UPR activation, a process deregulated during aging (Wang *et al*., 2014). Later, Wang *et al*. demonstrated that PERK is specifically activated in intestinal stem cells, having a functional role in promoting healthspan in flies. However, chronic engagement of this pathway becomes deleterious during aging in *Drosophila melanogaster* (Wang *et al*., 2015). A previous study also indicated that Xbp1 is both sufficient and required to limit intestinal stem cell proliferation (Wang *et al*., 2014). Recently, the same group reported that engagement of the Ire1/Xbp1 branch also results in lifespan extension in the same model under dietary restriction. The activation of Ire1/Xbp1 pathway in enterocytes under dietary restriction has a positive impact on lifespan of gut cells by regulating lipid synthesis (Luis *et al*., 2016). Overall, the functional significance of ER stress signaling to lifespan control has been inferred from several studies in simple model organisms.

## Cell‐nonautonomous control of organismal aging by the UPR

A novel concept is emerging based on research using fly and worm models of aging, indicating that the ER proteostasis network promotes health and lifespan through cell‐nonautonomous mechanisms, impacting whole organismal proteostasis (Mardones *et al*., 2015). Studies in *Caenorhabditis elegans* revealed that besides its importance in individual cells, the UPR acts as a key player in modulating global organism adaptability to stress during aging by integrating information at the level of the nervous system (Martinez *et al*., 2016a). Accordingly, UPR can be activated on a cell‐nonautonomous manner (Taylor & Dillin, 2013). The ectopic expression of XBP1s in neurons is able to engage a distal UPR activation in the intestine, thus increasing stress resistance and longevity in *Caenorhabditis elegans* (Taylor & Dillin, 2013). These results suggest that the nervous system may act as a central integrator and adjustor of global proteostasis, with possible major distal effects in the intestine. Importantly, other studies previously demonstrated that neuronal UPR regulates the innate immunity in the gut on a cell‐nonautonomous manner (Martinez & Hetz, 2012; Sun *et al*., 2012; Aballay, 2013). Chromatin remodeling factors in neurons can also engage ER stress responses through a cell‐nonautonomous mechanism (Kozlowski *et al*., 2014). Thus, accumulating evidence supports the idea that when an organism is exposed to environmental or pathogenic challenges, the ability of the nervous system to integrate these signals through the activation of the UPR favors the maintenance of homeostasis in various peripheral organs (Mardones *et al*., 2015). A similar model has been proposed for the heat‐shock response by Morimoto's group, where HSF‐1 in neurons regulates global responses to aging in the gut (Morley & Morimoto, 2004; van Oosten‐Hawle & Morimoto, 2014a; Douglas *et al*., 2015). Importantly, cell‐nonautonomous control of aging‐related pathways has been extensively described in different model organisms mediated by distinct signaling molecular mediators (Taylor *et al*., 2014; Leiser *et al*., 2015; Schinzel & Dillin, 2015). A recent study indicated that PERK is activated in intestinal stem cells by JAK/Stat signaling in response to ER stress in neighboring cells, regulating intestinal homeostasis and lifespan in flies (Wang *et al*., 2015). A cell‐nonautonomous mechanism has been also described in mammals, where overexpression of XBP1s in the hypothalamus modulates global energy balance through the propagation of signals to the liver and adipose tissue to adjust energy metabolism (Williams *et al*., 2014). Furthermore, the concept of ‘transcellular chaperone signaling’ was proposed in *Caenorhabditis elegans* where cells suffering stress from the accumulation of protein aggregates propagate signals to the neighbor tissue to induce adaptive responses and resist further damage (van Oosten‐Hawle & Morimoto, 2014b).

The molecular components that enable communication or signal propagation from neurons to the periphery are still unknown. It also remains to be established whether the activation of the UPR through cell‐nonautonomous mechanisms depends on the induction of ER stress in the target tissue or whether it is actually mediated by signaling events that engage UPR sensors in the absence of stress features (Mardones *et al*., 2015). This second possibility may be feasible since many examples are available showing that UPR stress sensors can be modulated by post‐translational modifications or protein–protein interactions in the absence of protein misfolding (Hetz, Chevet *et al*., 2015). Taylor and Dillin showed that the induction of cell‐nonautonomous UPR responses depends on the release of small clear vesicles from synaptic terminals of neurons expressing XBP1s (Taylor & Dillin, 2013). Additionally, XBP1s expression in neurons can promote the expression of brain‐derived neurotrophic factor (BDNF), which may in turn engage IRE1 signaling in a stress‐independent manner (Martinez *et al*., 2016b). Still, the specific mechanism of global proteostasis control in mammals and the neuronal circuits governing the propagation of UPR signals between cells remain to be determined. Although cell‐nonautonomous control of proteostasis has been validated in simple model organisms during aging, the occurrence of this process in mammals is still an open question. Overall, these examples illustrate how stress signals spread systemically in the organism to induce adaptive programs.

## ER stress response in mammalian aging

**Figure 2:**
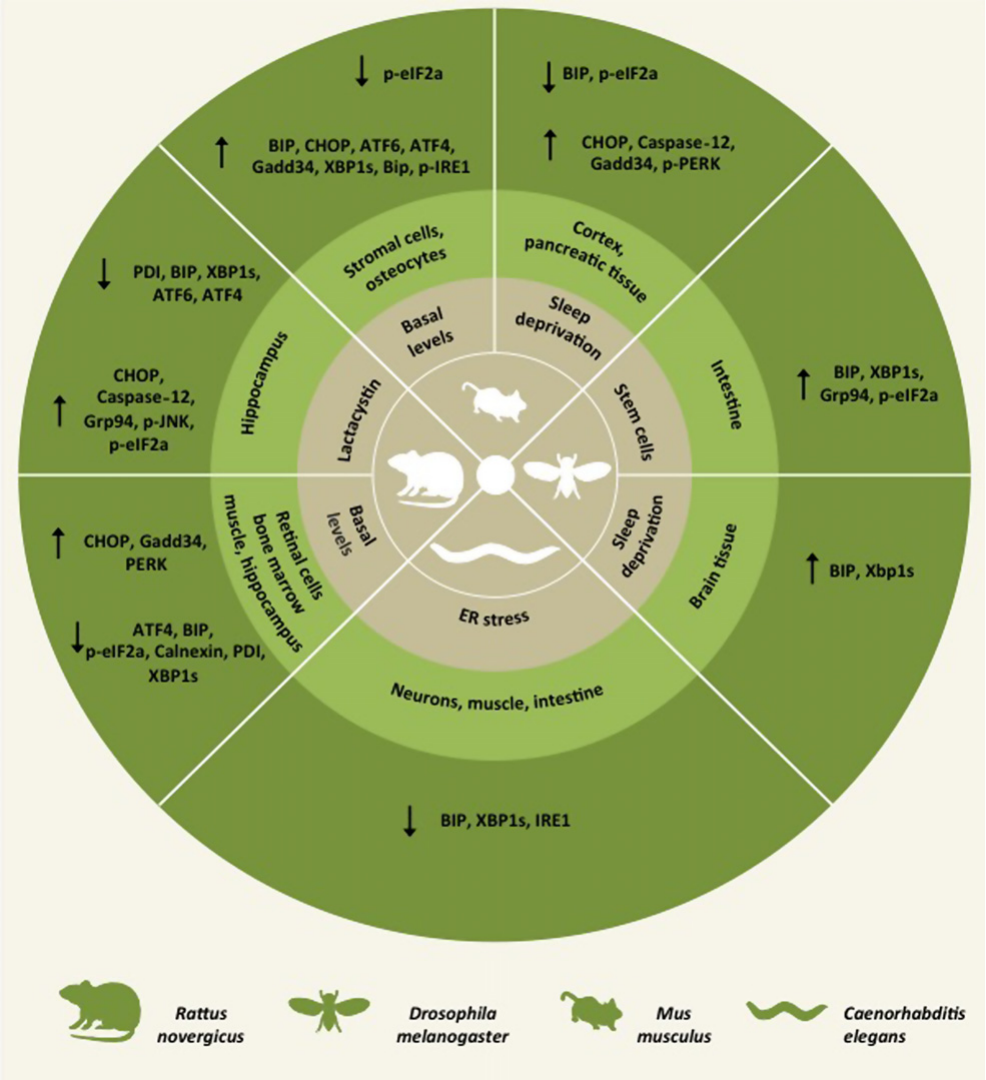
Endoplasmic reticulum (ER) stress in aging across species. The aging process is directly associated with a range of specific alterations in distinct components of the ER proteostasis network in different tissues, highlighting changes in components of the unfolded protein response and the folding machinery.

Although proteostasis impairment and ER stress are proposed as one of the hallmarks of aging, most of the studies addressing the contribution of the UPR to mammalian aging rely mostly on correlative data. For example, the expression of the ER chaperones BiP, calnexin, and PDI has been reported to be downregulated in the hippocampus of aged rats while pro‐apoptotic mediators such as CHOP and the ER‐located caspase‐12 are increased (Paz Gavilan *et al*., 2006; Gavilan *et al*., 2009). Additionally, another report demonstrated that levels of both ATF4 and BiP are decreased in this tissue (Hussain & Ramaiah, 2007). In aged brains, basal expression of PERK, GADD34, and total eIF2α is augmented contrasting with reduced levels of eIF2α phosphorylation (Hussain & Ramaiah, 2007). Moreover, young mice under sleep deprivation showed an increase in BiP and eIF2α phosphorylation, which was not observed in aged mice, but there was an upregulation of GADD34, CHOP, and caspase‐12 (Naidoo *et al*., 2008). Another study demonstrated similar observations in the pancreas of mice (Naidoo *et al*., 2014). Additionally, aged macrophages exhibit diminished IRE1 activation and increased susceptibility to ER stress‐dependent apoptosis (Song *et al*., 2013). These findings suggest that the ability to engage the UPR may be disrupted during aging; however, the functional significance of these observations is unknown (Fig. 2).

In contrast, several reports suggest that chronic ER stress is associated with aging in multiple tissues. Increased levels of CHOP, ATF4, and XBP1s were observed in primary osteocytes from aged mice when compared to adult mice exposed to ER stress‐inducing agents (Chalil *et al*., 2015). Similar results were reported in stromal cells from adipose tissue of aged mice, associated with augmented levels of BIP, CHOP, ATF6, and phosphorylated IRE1 (Ghosh *et al*., 2015). CHOP was also shown to be induced at baseline in muscular tissue of the hindlimb of aged rats (Baehr *et al*., 2016). Of interest, gene expression profile studies demonstrated that UPR‐target genes are one of the most affected pathways in bone marrow during aging (Kannan *et al*., 2016). At the level of the central nervous system, phosphorylation of CHOP, ATF6, GADD34, ATF4, and eIF2α are also upregulated in the retina of aged rats (Lenox *et al*., 2015).

ER stress signaling components have been shown to interact with classical aging‐related pathways suggesting a functional interconnection. For example, in the context of HD, we showed that XBP1 negatively regulates FOXO1 levels (Vidal *et al*., 2012). Several reports have also linked ER stress responses with the control of autophagy (reviewed in Vidal *et al*., 2014), a central pathway involved in proteostasis control (Kaushik & Cuervo, 2015). Insulin signaling is also linked to IRE1 function, as demonstrated in models of diabetes and obesity (Ozcan *et al*., 2004). Overall, these studies depict a general concept where mammalian aging is directly associated with the occurrence of chronic ER stress. Those findings may be explained by accumulative ER damage rather than an attenuation of UPR responses. Currently, functional analysis is needed to define the actual contribution of ER proteostasis to mammalian aging. Since a variety of mouse models are available to target specific UPR components in various tissues (Cornejo *et al*., 2013), the means to answer this fundamental question are already available.

## Aging as a risk factor to undergo neurodegeneration: a role of ER stress?

Abnormal aggregation of specific proteins is a hallmark of age‐related neurodegenerative diseases. Increasing evidence indicates that despite the fact that PMD‐related proteins distribute in different subcellular locations and have distinct binding partners, a common pathological consequence of their accumulation is the occurrence of ER stress. This mechanistic convergence is explained by the observation that disease‐related proteins actually disrupt the function of one or more components of the proteostasis network, highlighting the inhibition of ERAD, altered vesicle trafficking between the ER and Golgi, perturbed ER calcium homeostasis, autophagy dysregulation, and abnormal interactions with ER chaperones (Hetz & Mollereau, 2014; Vidal *et al*., 2014; Kaushik & Cuervo, 2015).

Importantly, with the exception of HD, familiar cases of PMDs account for less than 10% of cases, indicating that protein aggregation occurs in the absence of genetic mutations to the affected proteins. This observation suggests that alteration in different components of the proteostasis network during aging may contribute to protein aggregation. The involvement of ER stress in PMDs is highly complex, acting both as protective or detrimental (Hetz & Mollereau, 2014; Scheper & Hoozemans, 2015; Freeman & Mallucci, 2016). Indeed, the activity of the UPR in neurodegenerative diseases could either enhance or reduce neurodegeneration, depending on the process that is modulated by specific ER stress signals and the particular disease studied. A strong correlation between ER stress markers and signs of neurodegeneration has been reported in human postmortem tissue and animal models of PMDs. Remarkably, human neurons derived from induced pluripotent stem cells of AD, PD, and ALS patients revealed that ER stress is a prominent feature of this disease model (Chung *et al*., 2013; Kondo *et al*., 2013; Matus *et al*., 2014; Lee & Huang, 2017). Other studies also suggest that ATF4 may enhance axonal degeneration in AD through cell‐nonautonomous mechanisms (Baleriola *et al*., 2014; Wei *et al*., 2015). Importantly, the repressive effects of ER stress over protein synthesis were shown to contribute to the cognitive impairment observed in AD and PrD models by blocking the expression of synaptic proteins (Moreno *et al*., 2012, 2013; Freeman & Mallucci, 2016). Studies in *Drosophila melanogaster* (Loewen & Feany, 2010; Casas‐Tinto *et al*., 2011) and *Caenorhabditis elegans* (Safra *et al*., 2013) reported a functional role of XBP1 in neurodegeneration in AD. Interestingly, a polymorphism in the XBP1 promoter previously associated with bipolar disorders and schizophrenia (Kakiuchi *et al*., 2003; Du *et al*., 2008; Kim *et al*., 2009) was also pointed as a risk factor to develop AD in the Chinese population (Liu *et al*., 2013). In agreement with these findings, a new physiological function of XBP1 was proposed in the hippocampus in the control of learning and memory processes (Martinez *et al*., 2016b).

Genetic ablation of XBP1 in the nervous system uncovered a dynamic interconnection between the UPR and the autophagy pathway to handle protein aggregation. XBP1 deficiency protects against the development of experimental HD and ALS due to an increase in autophagy levels (Hetz *et al*., 2009; Vidal *et al*., 2012). In the context of PD, XBP1 deficiency also provided neuroprotection associated with the basal upregulation of several components of the ER proteostasis network, possibly reflecting the induction of nonlethal stress levels at the substantia nigra (Valdes *et al*., 2014). The concept of *hormesis* was proposed as an adaptive mechanism where a mild perturbation to neuronal proteostasis triggers compensatory mechanisms that enhance the capacity of the cell to cope with stress (Mollereau *et al*., 2016). In fact, treatment of animals with nonlethal doses of the ER stress agent tunicamycin (an inhibitor of N‐glycosylation) provides protection against PD possibly due to the induction of autophagy (Fouillet *et al*., 2012). Many other functional studies illustrate the therapeutic consequences of enforcing UPR adaptive outputs in ALS, PD, and HD (reviewed in Hetz & Mollereau, 2014; Freeman & Mallucci, 2016; Scheper & Hoozemans, 2015).

Recent evidence suggests that ER stress may underlay the differential neuronal vulnerability observed in neurodegenerative diseases, where most of the advances have been reported in ALS models (Rozas *et al*., 2016; Ruegsegger & Saxena, 2016). Disruption to the ER folding network is emerging as a key factor underlying the susceptibility of specific neuronal populations to undergo neurodegeneration (Filezac de L'Etang *et al*., 2015). In addition, genetic evidence has placed the ER proteostasis network in the etiology of ALS as mutations in two‐disulfide isomerase (PDIA1 and ERp57) were proposed as risk factors to develop ALS (Gonzalez‐Perez *et al*., 2015; Woehlbier *et al*., 2016). Alterations to the ER folding network may result in abnormal synthesis of synaptic proteins, having a negative effect on the integrity of neuromuscular junctions and neuronal connectivity (Bernard‐Marissal *et al*., 2012, 2015; Woehlbier *et al*., 2016). Similarly, genetic inactivation in BiP or its cofactor SIL1 results in spontaneous degeneration, leading to abnormal protein aggregation during aging (Zhao *et al*., 2005; Jin *et al*., 2014). Taken together, these studies suggest that alterations to the function of the ER during aging may contribute to synaptic dysfunction and abnormal protein aggregation, increasing the risk to develop neurodegenerative diseases.

## Concluding remarks

Imbalance of neuronal proteostasis is one of the pathological hallmarks of aging, and understanding its molecular defects will contribute to develop strategies to intervene age‐associated disorders. Because the nervous system is highly dynamic and plastic, the manifestation of clinical features in patients arises very late, after severe damage has already occurred. Likely, it is predicted that the development of strategies to improve the quality of the aging process will substantially reduce the probability to undergo PMDs. Despite the fact that proteostasis is composed of a complex network of individual interconnected signaling pathways, recent findings suggest that the maintenance of ER physiology is a prominent molecular target to prevent age‐related diseases affecting the nervous system. The involvement of ER stress in the biology of aging is complex as illustrated by most recent advances. The activity of the ER proteostasis network may not only operate as a mechanism to handle abnormal protein aggregation, but it is also proposed as an adjuster of brain function through fine‐tuning synaptic function. Specific neuronal populations are highly vulnerable to perturbations to ER function possibly because their metabolic state depends on the basal activity of the UPR. Furthermore, the UPR may orchestrate repair processes of the nervous system by controlling the expression of neurotrophins such as BDNF, and the regenerative capacity of axons and stem cells pools (Castillo *et al*., 2015; Martinez *et al*., 2016b; Onate *et al*., 2016). Regarding inflammatory reactions, the UPR is known to have important functions in macrophages and dendritic cells by modulating the secretion of pro‐inflammatory cytokines (Bettigole & Glimcher, 2015). In this context, future efforts should address the importance of the UPR to brain inflammation and the activity of astrocytes, microglia, and oligodendrocytes during aging. The fact that the UPR participates in the adjustment of energy and lipid metabolism, an additional layer of complexity, could be also explored to link the UPR with brain aging. Finally, the discovery of cell‐nonautonomous UPR responses and its relation to healthspan control adds a new concept as ER stress‐related signals in the brain may influence the capacity of the whole organism to adapt and cope with ER stress. All those aspects should be considered in future studies aiming to define the relative impact of ER stress on mammalian brain aging and its significance as a risk factor to develop neurodegenerative diseases.

Several novel small molecules are available to target selective UPR components and reduce ER stress levels (Table 2; Hetz *et al*., 2013; Maly & Papa, 2014; Gallagher & Walter, 2016; Gallagher *et al*., 2016; Axten, 2017), which promises possible new avenues to intervene the aging process. Importantly, some of these compounds have already been tested in preclinical models of PMDs (Table 2). However, it is important to consider possible side effects as the activity of the UPR has been linked to the physiology of many peripheral organs and the long‐term administration of UPR‐targeting drugs is predicted to induce liver failure, altered immune system function, pancreatic problems, among others maladies (Dufey *et al*., 2014). In this scenario, gene therapy is emerging as a strategy to locally reduce ER stress by delivering adaptive components of the UPR (i.e., XBP1s, BiP) specifically into the brain regions affected by distinct neurodegenerative diseases (Valenzuela *et al*., 2016). Overall, although the UPR is emerging as a central and evolutionarily conserved modulator of the normal process of aging, data available in mammalian systems are still correlative and remain to be functionally explored. As the UPR field has greatly evolved in the last five years in terms of generation of animal models and pharmacological tools, it is expected to witness future advances to underscore the significance of the UPR to brain aging and its relation to neurodegenerative diseases.

**Table 2:** Pharmacological modulation of the unfolded protein response (UPR). A summary is presented of chemically synthesized compounds to activate or inhibit the different UPR signaling components, including their efficacy in preclinical models of neurodegenerative diseases. Those components may emerge as candidates for lifespan/healthspan extension in the future (Maly & Papa, 2014; Gallagher *et al*., 2016; Gallagher & Walter, 2016; Plate *et al*. 2016; Axten, 2017)

UPR branch related	Drug	Molecular target	Effect	Readout	Model/disease
PERK	GSK2656157 and GSK2606414	PERK kinase domain	Inhibitor	Inhibition of eIF2α phosphorylation	Mouse/PrD, Tauopathies
	Integrated stress response inhibitor (ISRIB)	Guanine nucleotide exchange factor elF2B	Inhibitor	Decreased ATF4 expression	Mouse/PrD — memory and cognition
	Salubrinal	Binding GADD34 phosphatase complex	Inhibitor	Repression of translation, decrease in protein misfolding overload	Rat‐mouse/ALS, PD, Prion disease, spinal cord injury, multiple sclerosis, Charcot–Marie–Tooth 1B
	Guanabenz and Sephinl^1^	PPP1R15A elF2a phosphatase	Inhibitor	Repression of translation, decrease in protein misfolding overload	Mouse/ALS, Charcot–Marie–Tooth 1B
IRE1α	MKC‐3946 SFT‐083010	RNase active site	Inhibitor	Decrease of mRNA XBPI splicing	Mouse/cancer models
	Kinase inhibiting RNase attenuators 3 and 6 (Kira 3 and Kira 6)	Kinase domain	Inhibitor	Reduce IRE1α signaling	Mouse/diabetes and retinal damage
ATF6	Ceapin‐A1 Ceapin‐A7		Inhibitor	Inhibition of translocation of ATF6	Cells
	Small activators molecules		Activator	Induction of UPR‐regulated genes profile expression	Cells

## Funding

This work was directly funded by FONDAP program 15150012, US Office of Naval Research‐Global (ONR‐G) N62909‐16‐1‐2003, Millennium Institute P09‐015‐F, FONDEF ID16I10223, FONDEF D11E1007, U.S. Air Force Office of Scientific Research FA9550‐16‐1‐0384, CONICYT‐Brazil 441921/2016‐7, FONDECYT no 11160760 (CDA), and FONDECYT no 3150637 (GM). We also thank the support from ALS Therapy Alliance 2014‐F‐059, Muscular Dystrophy Association 382453, Michael J Fox Foundation for Parkinson's Research—Target Validation grant No 9277, FONDECYT no. 1140549, and ALSRP Therapeutic Idea Award AL150111 (CH).

## Conflict of interest

Authors declare that they have no conflict of interest.
